# Accumulation of Peptidoglycan *O*-Acetylation Leads to Altered Cell Wall Biochemistry and Negatively Impacts Pathogenesis Factors of Campylobacter jejuni*[Fn FN2]

**DOI:** 10.1074/jbc.M116.746404

**Published:** 2016-07-29

**Authors:** Reuben Ha, Emilisa Frirdich, David Sychantha, Jacob Biboy, Michael E. Taveirne, Jeremiah G. Johnson, Victor J. DiRita, Waldemar Vollmer, Anthony J. Clarke, Erin C. Gaynor

**Affiliations:** From the ‡ Department of Microbiology and Immunology, University of British Columbia, Vancouver, British Columbia V6T 1Z3, Canada,; the § Department of Molecular and Cellular Biology, University of Guelph, Guelph, Ontario N1G 2W1, Canada,; the ¶ Centre for Bacterial Cell Biology, Institute for Cell and Molecular Biosciences, Newcastle University, Newcastle upon Tyne NE2 4AX, United Kingdom, and; the ‖ Department of Microbiology and Immunology, University of Michigan Medical School, Ann Arbor, Michigan 48109

**Keywords:** acetylation, bacterial pathogenesis, cell wall, gram-negative bacteria, peptidoglycan, Campylobacter jejuni, O-Acetylation, O-Acetylesterase

## Abstract

*Campylobacter jejuni* is a leading cause of bacterial gastroenteritis in the developed world. Despite its prevalence, its mechanisms of pathogenesis are poorly understood. Peptidoglycan (PG) is important for helical shape, colonization, and host-pathogen interactions in *C. jejuni*. Therefore, changes in PG greatly impact the physiology of this organism. *O*-acetylation of peptidoglycan (OAP) is a bacterial phenomenon proposed to be important for proper cell growth, characterized by acetylation of the C6 hydroxyl group of *N*-acetylmuramic acid in the PG glycan backbone. The OAP gene cluster consists of a PG *O*-acetyltransferase A (*patA*) for translocation of acetate into the periplasm, a PG *O*-acetyltransferase B (*patB*) for *O*-acetylation, and an *O*-acetylpeptidoglycan esterase (*ape1*) for de-*O*-acetylation. In this study, reduced OAP in Δ*patA* and Δ*patB* had minimal impact on *C. jejuni* growth and fitness under the conditions tested. However, accumulation of OAP in Δ*ape1* resulted in marked differences in PG biochemistry, including *O*-acetylation, anhydromuropeptide levels, and changes not expected to result directly from Ape1 activity. This suggests that OAP may be a form of substrate level regulation in PG biosynthesis. Ape1 acetylesterase activity was confirmed *in vitro* using *p*-nitrophenyl acetate and *O*-acetylated PG as substrates. In addition, Δ*ape1* exhibited defects in pathogenesis-associated phenotypes, including cell shape, motility, biofilm formation, cell surface hydrophobicity, and sodium deoxycholate sensitivity. Δ*ape1* was also impaired for chick colonization and adhesion, invasion, intracellular survival, and induction of IL-8 production in INT407 cells *in vitro*. The importance of Ape1 in *C. jejuni* biology makes it a good candidate as an antimicrobial target.

## Introduction

*Campylobacter jejuni* is a leading bacterial cause of food-borne gastroenteritis in the developed world and the most common infectious antecedent to the autoimmune acute polyneuropathy Guillain-Barré syndrome (1, 2). As a commensal of the avian gut, it is a prevalent contaminant of uncooked poultry (3). Because of its high incidence rate, the costs of *C. jejuni* infection are a significant socioeconomic burden, making it both a health care concern and an economic issue (4). In addition, *C. jejuni* has been exhibiting alarming increases in resistance to ciprofloxacin and erythromycin, the most commonly used antibiotics for treatment of *C. jejuni* infection (5). Despite its prevalence, relatively little is known about *C. jejuni* pathogenesis in humans. Traditional virulence factors present in other gastrointestinal pathogens are either absent (*i.e.* dedicated type III secretion systems) or limited (*C. jejuni* possesses some stress-response elements such as the stringent response modulator SpoT, but it lacks several hallmark stress-response elements like RpoS and RpoE), or their role in pathogenicity is debated (*i.e.* the cytolethal distending toxin and a putative type IV secretion system on the pVIR plasmid) (6–11). However, factors considered to be fundamental aspects of bacterial physiology such as metabolism, stress response, and cell shape are hypothesized to play important roles in *C. jejuni* pathogenesis (12, 13).

The peptidoglycan (PG)^6^ sacculus is a heteropolymer of the bacterial cell wall composed of alternating β-1–4 *N*-acetylglucosamine (GlcNAc) and *N*-acetylmuramic acid (MurNAc) residues cross-linked to adjacent strands by peptides bound to the MurNAc residue. It is responsible for providing structural strength to the cell, enabling it to resist changes in osmotic pressure, and for maintenance of cell shape (14–16). The corkscrew motility of *C. jejuni* generated by its helical shape and polar flagella is thought to be important in enhancing its ability to move through viscous media, such as the mucous layer of the gastrointestinal tract (14, 17). Deletion of PG hydrolase enzymes Pgp1 and Pgp2 in *C. jejuni* has led to a change in morphology from helical to straight with accompanying defects in traits associated with pathogenesis, including motility in soft agar, biofilm formation, and chick colonization. PG isolated from Δ*pgp1* and Δ*pgp2* also exhibited altered stimulation of host cell NOD receptors, and Δ*pgp1* elicits an enhanced pro-inflammatory IL-8 response from INT407 epithelial cells upon infection (18, 19). Changes in PG biosynthesis and composition as well as the release of PG products have long been known to affect physiological and pathogenic properties of many bacterial species (20), including *Listeria monocytogenes* (21, 22), *Helicobacter pylori* (23), and *Streptococcus pneumoniae* (24). Current research continues to support this concept (15). Some recent studies have shown that changes in morphology and PG structure in *Mycobacterium tuberculosis* affect its physiology and virulence in mice (25); changes in morphology in *Proteus mirabilis* affect its swarming motility (26); and changes in morphology in *Helicobacter pylori* alter its motility and colonization potential (27, 28).

**FIGURE 1.**
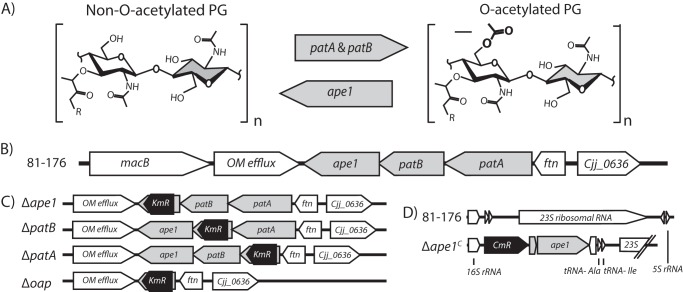
**Location of *O-*acetyl groups on peptidoglycan subunits, organization of the *C. jejuni* OAP gene cluster, and description of deletion mutant and complement construction.** *A,* structures of the disaccharide muropeptides showing non-*O*-acetylated PG and *O-*acetylated PG, location of *O-*acetylation (*arrow*), and the putative involvement of the *oap* genes. *B,* genomic organization of the *C. jejuni* OAP gene cluster in the 81-176 wild-type strain (*gray*). *cjj81176_0638*, *cjj81176_0639*, and *cjj81176_0640* are the *C. jejuni* homologs of Δ*ape1*, Δ*patB*, and Δ*patA* respectively, as identified by BLAST using the *N. gonorrhoeae* OAP genes sequences. *C,* OAP mutants were generated by homologous recombination with a mutated copy of the gene (or the entire cluster for Δ*oap*) in which a portion of the gene (or cluster) was deleted and replaced with a non-polar Km^R^ cassette (*aphA3*) (47). Resistance to Km was used as a selective marker for successful homologous recombination in *C. jejuni* with the mutated gene. *D,* complement construction (with Δ*ape1* used as an example, designated Δ*ape1^C^*). Each OAP gene plus upstream sequence containing the ribosomal binding site was cloned into the pRRC vector that contains homologous regions to three ribosomal intergenic regions downstream of the Cm^R^ cassette for selection of successful *C. jejuni* transformants. Complement constructs were transformed into their respective mutant backgrounds. *MacB*, macrolide-specific efflux pump; *OM efflux*, outer membrane efflux; *ftn,* ferritin; *23S*, 23S ribosomal RNA (48).

Understanding PG biosynthetic mechanisms in *C. jejuni* may prove advantageous to the development of new antimicrobials. It has been suggested that *O*-acetylation of peptidoglycan (OAP) machinery may be an attractive target (29–33). OAP occurs in both Gram-positive and Gram-negative bacteria and is characterized by the addition of an acetyl group to the C6 hydroxyl group of MurNAc in the PG glycan backbone (Fig. 1*A*). This modification confers resistance to lysozyme (34, 35), which cleaves β-1,4-glycosidic bonds between MurNAc and GlcNAc (36). Despite the intrinsic resistance to lysozyme provided by the outer membrane of Gram-negative bacteria, lysozyme resistance was shown to be important in *H. pylori* using mutants defective in OAP addition and similar glycan modifications (37). These strains showed increased susceptibility to physiologically relevant concentrations of lysozyme in the presence of the host lactoferrin, which has membrane permeabilization properties (38, 39). *O-*Acetylated gonococcal PG is implicated in the development of arthritic symptoms and is hypothesized to be attributable to increased PG hydrolase resistance leading to large fragments of circulating PG (40). In addition, OAP is believed to be involved in the regulation of PG turnover by inhibiting endogenous lytic transglycosylase (LT) activity. LTs require an unmodified MurNAc C6 hydroxyl moiety to cleave β-1,4-glycosidic bonds between MurNAc and GlcNAc, generating anhydromuropeptides (anhMPs). LTs are therefore important for generating insertion sites for newly synthesized muropeptides during cell growth and division (41).

The OAP gene cluster was initially identified in *Neisseria gonorrhoeae* and was found to be responsible for OAP in many Gram-negative pathogens (42). It consists of a putative transmembrane protein, PG *O-*acetyltransferase A (PatA) predicted to be involved in the translocation of acetyl moieties from a cytoplasmic source into the periplasm, a periplasmic transferase, PG *O-*acetyltransferase B responsible for *O-*acetylation of MurNAc, and a periplasmic *O-*acetylpeptidoglycan esterase (Ape1) for MurNAc de-*O*-acetylation (Fig. 1*A* and *B*) (42–44). Since their discovery, PatB and Ape1 from *N. gonorrhoeae* and *Neisseria meningitidis* have been well characterized biochemically, including descriptions of the catalytic residues, mechanism of enzyme activity, and substrate specificity (30, 32, 43, 45). The availability of a recently solved crystal structure for *N. meningitidis* Ape1, recently identified Ape1 inhibitors, and studies showing reduced septicemia in mice infected with *N. meningitidis ape1* mutants lend support for the application of Ape1 as an antimicrobial target (31, 33, 46).

*C. jejuni* encodes previously unstudied homologs of the OAP genes *patA*, *patB,* and *ape1*. In this work, the roles of these genes in PG *O-*acetylation and overall PG biosynthesis, as well as biological and pathogenic attributes were assessed via construction of strains deleted for each or all of these genes followed by biochemical and phenotypic analyses. Each mutant exhibited changes in PG *O-*acetylation consistent with predicted gene product functions. The accumulation of *O-*acetylated PG was found to be detrimental to *C. jejuni* fitness, whereas diminished *O-*acetylation had little to no effect. Δ*ape1* exhibited defects in PG muropeptide composition, cell morphology, pathogenic attributes, and host-pathogen interactions, whereas Δ*patA*, Δ*patB,* and Δ*oap* mutants exhibited no or, in rare cases, only minimal defects for these phenotypes.

## Results

### 

#### 

##### C. jejuni OAP Genes Were Identified by BLAST and Mutant and Complemented Strains Were Generated

The OAP gene cluster was identified in *C. jejuni* 81-176 wild type by BLAST analysis using the *N. gonorrhoeae* OAP gene sequences. The loci identified were *cjj81176_0640, cjj81176_0639,* and *cjj81176_0638* for *patA*, *patB*, and *ape1*, respectively (Fig. 1*B*). Amino acid sequence identity and sequence similarity for these genes were 35/53%, 39/57%, and 26/44% identity/similarity to *N. gonorrhoeae patA*, *patB*, and *ape1*, respectively.

To investigate the role of OAP in *C. jejuni*, the *patA, patB,* and *ape1* homologs, as well as the entire gene cluster, were inactivated by deletion-insertion mutagenesis with the non-polar Km^R^ cassette (*aphA-3*) from pUC18K-2 lacking a transcriptional termination site (Fig. 1*C*; supplemental text S1) (47). Complementation was achieved using the pRRC integration vector (48). For complementation, the coding region of each OAP gene plus upstream sequence containing the ribosomal binding site was inserted into the genome of the corresponding mutant at ribosomal intergenic regions along with a Cm^R^ cassette (the Δ*ape1* complemented strain, designated Δ*ape1^C^*, is shown in Fig. 1*D* as an example). Expression of the wild-type genes at the rRNA site was driven from the promoter of the Cm^R^ cassette.

Growth rate experiments performed on all mutant strains indicated no significant difference in growth rates in broth cultures up to 36 h (data not shown) with the exception of Δ*ape1^C^*, which grew at a slower rate (although this slower growth rate did not affect the ability of Δ*ape1^C^* to complement Δ*ape1* phenotypes). Differences were observed in the long term survival properties of Δ*ape1* with more modest differences exhibited by Δ*patA*, Δ*patB,* Δ*oap,* and Δ*ape1^C^.* At 48 h, a 1.0-log increase in recovery was observed for Δ*ape1,* but recovery fell at 72 h by 0.8-log relative to wild type. At 48 h, Δ*patA* and Δ*patB* exhibited a 0.3-log increase, and Δ*oap* showed a 0.4-log increase in recovery, relative to wild type. The recovery of all three OAP-deficient mutants was 0.3-log lower than wild type at 72 h. Δ*ape1^C^* exhibited a 0.8-log increase in recovery at 48 h and a 0.3-log decrease at 72 h relative to wild type (data not shown).

##### O-Acetylation Levels of Purified PG from Deletion Mutants Reflect the Putative Functions of the C. jejuni OAP Gene Cluster

To determine whether the *C. jejuni* OAP gene homologs were involved in OAP, PG *O-*acetylation levels were determined for the mutants of the three putative OAP genes (Δ*patA*, Δ*patB*, and Δ*ape1*) and for the mutant lacking the entire cluster (Δ*oap*) (Fig. 1*C*). PG was isolated from strains using an established protocol that minimizes spontaneous *O-*linked acetate hydrolysis and was assessed for OAP levels by quantifying released acetate and MurNAc (49, 50).

The *O-*acetylation level for the wild-type strain 81-176 was determined to be 12.5 ± 0.71% relative to MurNAc content. *O-*Acetylation levels among the mutants varied according to their predicted function (Table 1). Deletion of *patA* and *patB* resulted in a reduction in *O-*acetylation levels at 2.45 ± 0.14 and 3.05 ± 0.22% relative to MurNAc content, respectively. Deletion of the entire gene cluster in Δ*oap* resulted in a decrease in *O-*acetylation levels to 2.10 ± 0.18%, similar to that of Δ*patA* and Δ*patB.* Previous studies using *Escherichia coli* (which lacks PG *O-*acetyl groups) showed undetectable levels of acetate using identical methods (50, 51). This suggests that *patA/B* contributes to PG *O-*acetylation in *C. jejuni,* but their absence is insufficient to abolish OAP. Deletion of *ape1* led to an increase in *O-*acetylation to 35.6 ± 2.25% relative to total MurNAc content. These results are in accordance with the functions described for homologs in *N. gonorrhoeae* and *N. meningitidis* (30, 42–44). *O-*Acetylation levels were restored to wild-type levels in the Δ*ape1* complement (11.78 ± 0.52%). Analysis of the *O-*acetylation levels for Δ*patA* and Δ*patB* complements were not performed as, unlike the Δ*ape1* mutant, phenotypic differences between these mutants, Δ*oap* and wild type, were in almost every case not statistically significant or were minimal (see below).

**TABLE 1:** ***O*-Acetylation levels of *C. jejuni* 81-176, Δ*ape1,* Δ*ape1^C^* (complemented Δ*ape1*), Δ*patB,* Δ*patA,* and Δ*oap* (a mutant in which the entire cluster was deleted: *ape1, patB,* and *patA*), as determined by base-catalyzed hydrolysis and release of acetate reported as a % *O*-acetylation relative to Mur*N*Ac content**

Strain	% *O*-acetylation*^a^* (mean ± S.D.)
81-176	12.5 ± 0.71
Δ*ape1*	35.6 ± 2.25
Δ*ape1^C^*	11.8 ± 0.52
Δ*patB*	3.05 ± 0.22
Δ*patA*	2.45 ± 0.14
Δ*oap*	2.10 ± 0.18

*^a^* Results shown are of one representative biological replicate measured in triplicate ± S.D.

##### C. jejuni OAP Mutants Exhibit Altered PG Muropeptide Profiles, with Δape1 Displaying the Most Dramatic Changes from Wild Type

**FIGURE 2.**
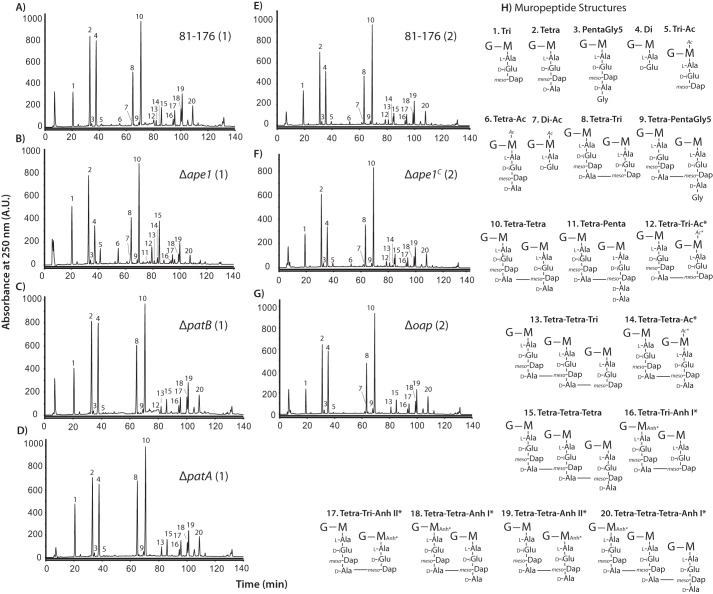
**HPLC elution profile of *C. jejuni* muropeptides and proposed muropeptide structures.** Purified PG was digested with cellosyl, and the resulting muropeptides were reduced with sodium borohydride and separated on a Prontosil 120-3-C18 AQ reverse-phase column. HPLC profiles are shown for wild-type strain 81-176 (*A* and *E*), Δ*ape1* (*B*), Δ*patB* (*C*), Δ*patA* (*D*), Δ*ape1^C^* (*F*), and Δ*oap* (*G*). Muropeptide profiles were generated in two sets of experiments indicated by (*1*) for Sample Set 1 and (*2*) for Sample Set 2. The muropeptide structure represented by each peak was determined previously by mass spectroscopy (18), and proposed muropeptide structures of each peak corresponding to the peak number in the chromatogram are shown in *H*. The summary of the muropeptide composition is shown in Table 3. *G*, *N*-acetylglucosamine; *M*, reduced *N*-acetylmuramic acid; *l**-Ala*, l-alanine; *d**-iGlu*, d-isoglutamic acid; *meso-Dap*, *meso*-diaminopimelic acid; *d*-*Ala*, d-alanine *Ac*, *O-*acetyl groups at MurNAc C6 position; *Anh*, 1,6-anhydro group of MurNAc; *, it is not known on which MurNAc residue the modification occurs.

*O*-Acetylation has been described as a PG maturation event occurring after transglycosylation and transpeptidation (30). Cleavage of PG by bacterial LTs is inhibited by PG *O-*acetylation. Thus, *O-*acetylation may impact PG maturation events in *C. jejuni*, affecting aspects such as muropeptide profiles and glycan chain length (52). To investigate this, PG was isolated from wild type and each of the mutant and complemented strains, and the muropeptide composition was determined. HPLCs are shown in Fig. 2. Raw data (relative abundance of each muropeptide) and summarized muropeptide profiles can be found in Tables 2 and 3, respectively.

**TABLE 2:** **Muropeptide composition of *C. jejuni* wild-type 81-176, Δ*ape1*, Δ*patB*, and Δ*patA*, Δ*ape1^C^*, Δ*patB^C^*, Δ*patA^C^*, and Δ*oap* showing relative abundance of muropeptides corresponding to peaks in HPLCs (Fig. 2)**

Peaks*^a^*	Muropeptide	Sample Set #1				Sample Set #2				
		*81-176*	Δ*ape1*	Δ*patB*	Δ*patA*	*81-176*	Δ*ape1^C^*	Δ*patB^C^*	Δ*patA^C^*	Δ*oap*
1	Tri	5.8	12.3	7.3	9.5	8.8	9.6	8.8	6.0	6.2
*^b^*	Tetra-Gly-4					0.4	0.4	0.4	0.3	0.4
2	Tetra	16.1	16.6	15.8	15.2	16.1	16.9	16.2	15.7	15.1
3	Penta-Gly-5	0.6	0.8	0.7	0.7	0.8	1.0	1.2	0.9	1.0
4	Di	16.3	8.3	16.1	14.3	13.0	10.1	10.4	16.2	15.7
5	Tri-Ac	0.3	2.8	0.3	0.3	1.0	1.2	0.7	0.5	0.4
6	Tetra-Ac	0.6	2.6	0.0	0.0	0.6	0.7	0.5	0.0	0.0
7	Di-Ac	0.0	1.9	0.0	0.0	0.2	0.5	0.5	0.6	0.4
8	TetraTri	9.7	7.6	11.1	13.7	10.5	10.1	10.6	11.5	11.2
9	TetraPenta-Gly-5	0.4	0.7	0.6	1.0	0.8	0.9	1.0	0.8	1.2
10	TetraTetra	19.0	17.0	18.0	21.2	21.4	22.4	23.0	22.4	22.5
11	TetraPenta	0.0	0.1	0.0	0.0					
12	TetraTri-Ac	1.8	3.4	1.6	0.0	1.5	1.7	0.9	0.0	0.0
13	TetraTetraTri	0.8	0.5	0.9	1.5	1.0	0.9	1.3	1.3	1.4
14	TetraTetra-Ac	0.3	1.1	0.0	0.0	1.7	1.8	1.4	0.4	0.0
15	TetraTetraTetra	2.9	7.0	1.9	2.8	2.5	2.8	3.1	2.9	3.4
16	TetraTriAnh I	1.2	0.5	1.3	1.3	1.1	1.0	1.1	1.3	1.2
17	TetraTriAnh II	2.6	1.6	2.5	2.7	2.4	2.1	2.2	2.5	2.5
18	TetraTetraAnh I	3.0	1.8	2.8	2.7	2.8	2.8	3.0	3.1	3.0
19	TetraTetraAnh II	5.6	3.7	4.9	4.9	5.2	5.0	5.1	5.4	5.5
*^b^*	TetraTetraTriAnh					1.1	0.9	1.3	1.4	1.7
20	TetraTetraTetraAnh	4.3	1.9	3.9	4.3	4.1	4.2	4.9	4.5	5.3
1–20	All known*^c^*	91.2	92.3	89.5	95.9	96.8	97.1	97.4	97.7	97.9

*^a^* Peak numbers correspond to those from HPLCs in Fig. 2. Muropeptides are named according to Glauner *et al.* (88) and are depicted in Fig. 2*H*. Di, disaccharide dipeptide (disaccharide = β1,4-linked *N*-acetylglucosamine-*N*-acetylmuramic acid); Tri, disaccharide tripeptide; Tetra, disaccharide tetrapeptide; Penta, disaccharide pentapeptide Muropeptides 1–7 are monomeric, and muropeptides 8–20 are dimers and trimers connected by peptide cross-links. Modifications: Gly, glycine in position 5 of a peptide side chain; Ac, *O*-acetyl groups at the C-6 hydroxyl group of MurNAc; Anh, 1,6-anhydro-*N*-acetylmuramic acid.

*^b^* Peak was previously unidentified in Sample Set #1.

*^c^* The total abundance does not add up to 100% due to the presence of peaks for which a structure has not been assigned.

**TABLE 3:** **Summary of PG muropeptide composition for *C. jejuni* 81-176, Δ*ape1*, Δ*patB,* Δ*patA,* Δ*ape1^C^*, Δ*patB^C^,* Δ*patA^C^*, and Δ*oap***

Muropeptide species	Percentage of peak area*^a^*								
	Sample Set #1				Sample Set #2				
	*81-176*	Δ*ape1*	Δ*patB*	Δ*patA*	*81-176*	Δ*ape1^C^*	Δ*patB^C^*	Δ*patA^C^*	Δ*oap*
Monomers (total)	43.5	49.1	44.9	41.7	42.2	41.5	39.6	41.2	40.0
Dipeptide	17.8	11.0*	18.0	15.0	13.6	11.0	11.2	**17.2**	**16.4**
Tripeptide	6.7	16.4*	**8.5**	10.2*	10.1	11.1	9.7	6.6*	6.7*
Tetrapeptide	18.3	20.9	17.6	15.8	17.2	18.1	17.1	16.1	15.5
Pentapeptides-Gly	0.6	0.9*	0.7	0.7	0.8	**1.0**	1.3*	0.9	**1.0**
*O*-Acetylated	1.0	8.0*	0.3*	0.3*	1.9	**2.4**	1.7	1.1*	0.8*
Dimers (total)	47.7	40.7	47.6	49.4	48.8	49.4	49.5	48.5	48.1
TetraTri	16.8	14.2	18.4	18.5	16.0	15.5	15.2	15.7	15.2
TetraTetra	30.5	25.6	28.6	29.9	32.0	33.0	33.2	32.0	31.7
TetraPentaGly	0.4	0.9*	0.6*	1.0*	0.8	0.9	1.1*	0.8	1.2*
Anhydro-Dimers	13.5	8.2*	12.7	12.1	11.8	11.3	11.6	12.6	12.4
*O*-Acetylated	2.2	4.9*	**1.7**	0*	3.2	3.7	**2.3**	0.4*	0*
Trimers (total)	8.8	10.2	7.5	8.8	7.8	8.1	**9.5**	8.9	10.2*
TetraTetraTri	0.9	0.5*	1.0	1.5*	1.0	0.9	1.3*	1.3*	1.4*
TetraTetraTetra	8.0	**9.7**	6.5	7.3	6.8	7.2	**8.2**	7.6	**8.8**
Dipeptides (total)	17.8	11.0*	18.0	15.0	13.6	11.0	11.2	**17.2**	**16.4**
Tripeptides (total)	15.4	23.6*	18.0	**19.9**	18.4	19.1	17.7	14.9	**14.7**
Tetrapeptides (total)	65.9	64.0	62.9	63.9	65.1	67.1	67.6	64.8	65.1
Pentapeptides (total)	0.8	1.3*	**1.0**	1.2*	1.2	1.5	**1.9**	1.3	1.6*
*O*-Acetylated (total)	2.1	10.4*	1.2*	0.3*	3.5	4.3	2.9	1.3*	0.8*
Anhydromuropeptides	8.3	4.8*	7.8	7.5	7.7	7.4	7.9	8.3	8.6
Average chain length	12.0	20.8*	12.8	13.3	13.0	13.5	12.6	12.0	11.6
Degree of cross-linkage	29.7	27.2	28.8	30.6	29.6	30.1	31.1	30.2	30.9
% peptide cross-links	56.5	50.9	55.1	58.3	57.8	58.5	60.4	58.8	60.0

*^a^* Values represent the percentage area of each muropeptide from raw data (found in Table 2) calculated to give a total of 100%. Boldface numbers represent a change in relative abundance of ≥20% from wild type. Boldface numbers with an asterisk represent ≥30% change from wild type. Percentages shown are calculated from values rounded to the nearest 0.1%. Muropeptide profiles were generated in two sets of experiments, Sample Set #1 and Sample Set #2. Muropeptide profiles were compared with the wild-type 81-176 muropeptide profile that was analyzed in the same sample set.

The method used for muropeptide analysis results in the loss of some of the PG *O-*acetyl groups (due to the alkaline conditions for NaBH_4_ reduction resulting in base-catalyzed hydrolysis of the *O-*linked acetate) and is thus less precise at determining *O-*acetylation levels than the methodology used above. Nonetheless, similar trends were observed, further supporting gene product function. PG *O-*acetylation levels were reduced in Δ*patA*, Δ*patB*, and Δ*oap* and increased in Δ*ape1* relative to wild-type 81-176. Monomeric *O-*acetylated tetrapeptide species and *O-*acetylated tetra-tetra dimeric species were absent in Δ*patB*, Δ*patA*, and Δ*oap*. The abundance of all detectable *O-*acetylated muropeptide species was increased in Δ*ape1.*

Δ*ape1* exhibited a large decrease in total anhMP species and an increased average glycan chain length compared with wild type, similar to observations made in *N. meningitidis* (33), and is consistent with the observed *O-*acetylation levels, as de-*O*-acetylation must precede LT activity. Conversely, in the absence of *patA* or *patB*, the relative abundance of anhMP species did not vary strongly from wild type.

Changes were also observed in other muropeptide species between wild type and Δ*patA*, Δ*patB*, Δ*oap,* and Δ*ape1*. The Δ*patA* mutant showed an increase in monomeric tripeptides, tetra-penta dipeptides, and tetra-tetra-tri tripeptides. For Δ*patB*, the monomeric tripeptides and dimeric tetra-penta species increased relative to wild type. Δ*oap* exhibited some differences from wild type that followed the same trend as with Δ*patA,* an increase in tetra-penta dipeptides and tetra-tetra-tri tripeptides, and some that were unique to Δ*oap*, an increase in dipeptides and a decrease in tripeptides. Analyses of PG muropeptide profiles for Δ*patA-* and Δ*patB-*complemented strains Δ*patA^C^* and Δ*patB^C^* showed minimal changes from wild type with restored *O-*acetylation levels for Δ*patB^C^* but not Δ*patA^C^*. The largest number of changes and greatest degree of change occurred in Δ*ape1*. In Δ*ape1*, total dipeptide species decreased and total tripeptides and pentapeptides increased relative to wild type. The total amount of dimers also appeared to be slightly lower in Δ*ape1*. The majority of the muropeptide changes was restored to near wild-type levels in the Δ*ape1* complement strain (Δ*ape1^C^*).

##### Recombinant Ape1 Has in Vitro Acetylesterase Activity on p-Nitrophenyl Acetate and O-Acetylated PG

**FIGURE 3.**
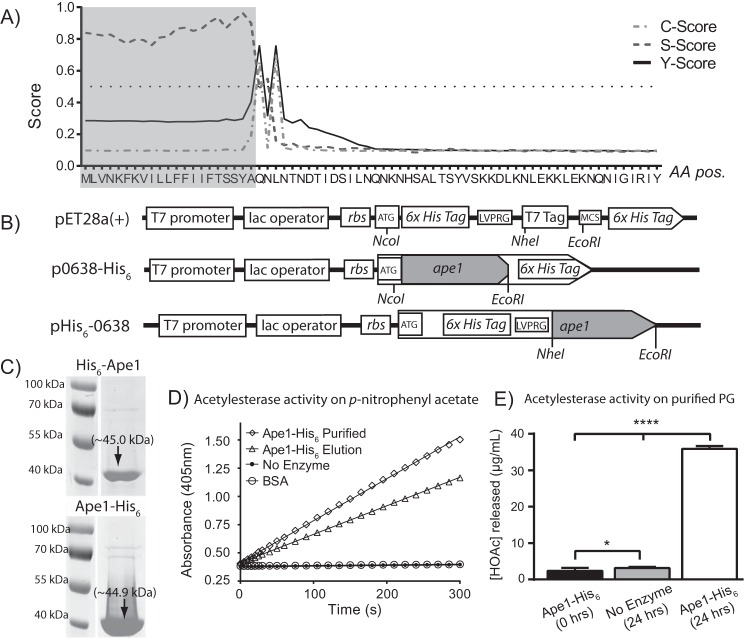
*A,* SignalP 4. 1 server (83) output for signal peptide prediction of in-frame translation of *Cjj-81-176_0638. C-score* (the predicted first amino acid of the mature protein), *S-score* (the likelihood that a particular amino acid is part of a signal peptide), and *Y-score* (amino acid with a high C-score exhibiting the greatest change in the S-score) predicted the cleavage site to be between the 21st and 22nd amino acids. *B, Cjj81-176_0638* was cloned in-frame without the signal peptide into pET28a(+) protein expression vectors. *Top,* map of cloning sites in pET28a(+) commercial expression vector. *Middle,* Ape1-His_6_ expression construct. NcoI and EcoRI were used to produce a C-terminal His_6_-tagged Ape1 protein that uses ATG start codon and TGA stop codon encoded in the vector. *Bottom,* His_6_-Ape1 expression construct. NheI and EcoRI were used to produce an N-terminal His_6_-tagged protein that uses AUG start codon encoded by the vector and the original stop codon from *Cjj81-176_0638. rbs*, ribosome-binding site; *LVPRG,* thrombin cleavage site; *MCS*, multiple cloning sites. Gene organization not to scale. *C,* His_6_-tagged Ape1 after nickel-nitrilotriacetic acid-agarose purification shows protein of the predicted size (45.0 and 44.9 kDa for His_6_-Ape1 and Ape1-His_6_ respectively) in eluted fractions after SDS-PAGE analysis. *D,* purified Ape1-His_6_ exhibits acetylesterase/deacetylase activity using pNPAc as a substrate (45). Reactions were monitored over 5 min as a change in the absorbance at 405 nm (formation of *p*-nitrophenol) after cleavage of the acetyl group. No enzyme control and BSA control are overlapping and show no acetylesterase activity. Results shown are from one protein purification experiment. Results are reproducible for each expression and purification experiment, and activity was routinely assessed before performing enzymatic assays on PG. *E,* Ape1-His_6_ has acetylesterase activity using PG muropeptides as a substrate. Determination of acetic acid concentration after treatment of Δ*ape1* PG with Ape1-His_6_ for 24 h was performed using Megazyme acetic acid assay kit. Treatment and no enzyme control were compared with acetic acid concentration at 0 h of treatment using Student's *t* test with * and **** indicating *p* values of <0.05 and <0.0001, respectively. Results are from one representative experiment of two biological replicates performed in triplicate.

Based on the muropeptide data observed for Δ*ape1* and phenotypic data shown below, the acetylesterase activity of Ape1 was confirmed biochemically. Ape1 was expressed with a His_6_ tag and minus the signal peptide at either the N or C terminus and purified (Fig. 3, *A* and *B*). Both recombinant proteins purified well (Fig. 3*C*), producing 3 ml of 0.9–1.2 mg/ml protein after dialysis from a starting culture of 100 ml. The expected sizes of the recombinant proteins are 45.0 and 44.9 kDa for His_6_-Ape1 and Ape1-His_6_, respectively. The specific activity of purified Ape1 was determined using *p*-nitrophenylacetate (*p*NPAc), a common substrate used to test esterase activity (31, 32). Specific activity for Ape1-His_6_ ranged between 26.1 and 38.9 μmol/min/mg of protein (Fig. 3*D*), which was higher than the reported specific activity of ∼10.4 μmol/min/mg for *N. gonorrhoeae* Ape1 measured under similar reaction conditions (32). This demonstrates that the recombinant protein exhibits acetylesterase activity. His_6_-Ape1 showed similar specific activity with *p*NPAc (data not shown).

Ape1-His_6_ was also assayed for acetylesterase activity on its native substrate (Fig. 3*E*), *O-*acetylated PG. PG isolated from Δ*ape1* was used as the substrate due to the increased PG *O-*acetylation levels in this strain. Cleavage of *O-*acetyl groups was assessed using a commercial acetic acid assay kit (Megazyme) as an end point experiment. At 0 h, the average acetate concentration in the sample was 2.4 ± 0.3 μg/ml. Incubation of Δ*ape1* PG for 24 h in the absence of enzyme resulted in an average acetate concentration of 3.1 ± 0.1 μg/ml, and an average acetate concentration of 35.9 ± 0.7 μg/ml after incubation with Ape1-His_6_. Because of the insoluble nature of PG, data from this assay cannot be expressed in the classical definitions of enzyme kinetics using the native substrate.

##### Microscopy and CellTool Analyses of C. jejuni Δape1 Population Morphology Reveal Shape Pleomorphism

**FIGURE 4.**
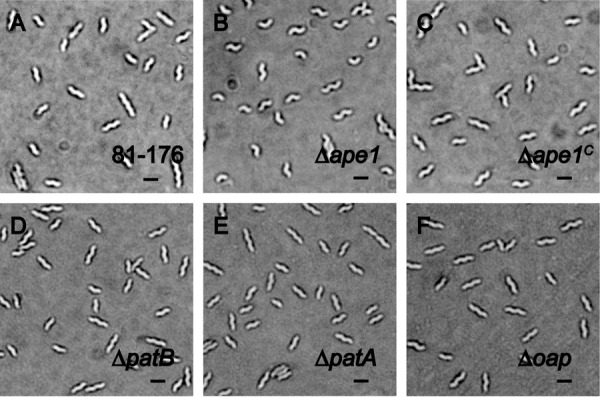
***C. jejuni* Δ*ape1* mutant has a pleomorphic cell shape, and other OAP mutants display unaltered cell morphology.** DICM showing the morphology of wild-type strain *C. jejuni* 81-176 (*A*), the differentially curved Δ*ape1*strain (*B*), the complemented strain Δ*ape1^C^* with restored morphology (*C*), Δ*patB* (*D*), Δ*patA* (*E*), and Δ*oap* (*F*). Cells were harvested from 7 h of growth in MH-TV broth at a mid-exponential phase of growth. Scale is 2 μm (*black bar*).

Because a number of changes were observed in the muropeptide profiles of the OAP mutants, it was hypothesized that these changes may result in changes in cell shape. The morphology was examined by DICM after 4 and 7 h of growth initiated at an *A*_600_ of 0.05 to obtain early- and mid-exponential phase cultures (Fig. 4). Whereas wild type exhibited the classical *C. jejuni* helical shape (Fig. 4*A*), Δ*ape1* exhibited primarily “comma-shaped” and differentially curved cells (Fig. 4*B*). Wild-type helical morphology was restored upon complementation (Fig. 4*C*). A distinct change in morphology was not observed for the other OAP mutant populations (Fig. 4, *D–F*).

**FIGURE 5.**
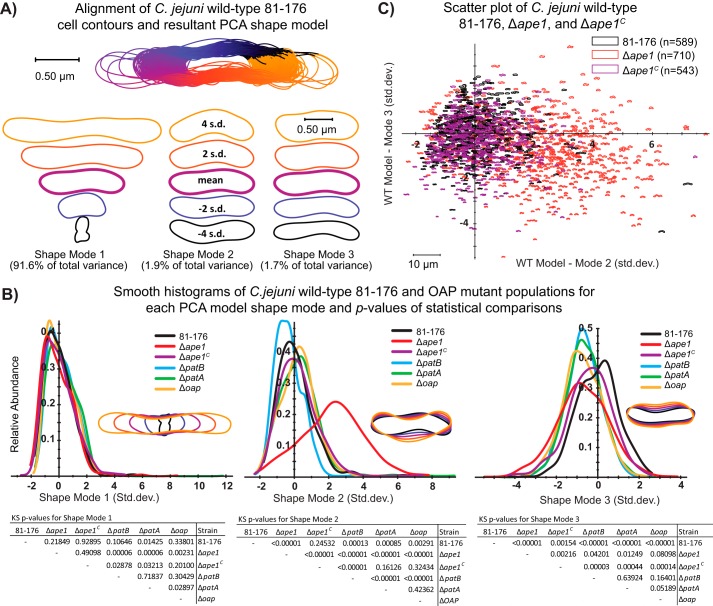
**CellTool analysis of wild-type strain 81-176, Δ*ape1*, Δ*ape1^C^*, Δ*patB*, Δ*patA*, and Δ*oap* population morphology.** Differential interference contrast images were taken of strains grown for 7 h in MH-TV broth at a starting *A*_600_ of 0.05 (to mid-exponential phase). Images were converted to binary format (*white cells* on a *black background*), and lumps and artifacts were manually removed before processing with CellTool “extract contours function” to generate contours representing each cell (53). *A,* contour extraction, alignment, and generation of the PCA shape model for *C. jejuni* wild-type strain 81-176. CellTool “align contours” function was used to align the contours of the wild-type population to one another. *B,* PCA was performed to generate a wild-type shape model that explains 95% variation in the population in principal components called “shape modes.” Shape modes 1, 2, and 3 represent variation in length, curvature/wavelength, and width, respectively. The extracted contours of the mutant populations were then aligned to the wild-type shape model, and a measurement representing the normalized standard deviation from the wild-type mean in each shape mode was generated and depicted graphically. KS tests were performed for each shape mode between each population and are summarized below the plots. *C,* measurements of wild type, Δ*ape1*, and Δ*ape1^C^* were plotted with shape mode 2 along the *x* axis and shape mode 3 along the *y* axis to create a scatterplot showing the variation in the different populations.

The open-source shape analysis program CellTool (53) was used to quantify the changes in shape in the OAP mutants (Fig. 5). The program contains a set of tools used to extract shapes from binary images that can then be used to assess and compare the population morphology using a variety of metrics. Extracted shapes from the wild-type population were aligned to one another, and principal component analysis (PCA) was performed to generate a baseline model for variation within the wild-type population. At mid-exponential phase, three shape modes (principal components that define specific changes in cell shape in the population) described 95% of the morphological variation in the *C. jejuni* wild-type population. Each shape mode represented an observable metric (Fig. 5, *A* and *B*). Shape mode 1 corresponded to variation in cell length, explaining >90% of the variance (as expected because a population likely exhibits asynchronous growth and division). Shape mode 2 explained 1.9% of the variance and appeared to have some relation to the curvature and wavelength of the cell. A third shape mode explained 1.7% of the variance and described differences in cell width. Contours of mutants were aligned to the wild-type shape model as a reference and Kolmogorov-Smirnov (KS) statistical tests were used to compare sample probability distributions. Based on the large population of bacterial cells assessed and conditions required for KS analysis, a *p* value of 0.00001 was used as a cutoff for significance (Fig. 5*B*) (54).

No strains were significantly different from the wild type in shape mode 1 (cell length) or from each other, with the graphical output also showing that the population distributions overlay very closely. In shape mode 2 (cell curvature), some differences in population distribution between wild type and Δ*ape1^C^*,Δ*patA*, Δ*patB*, and Δ*oap* were significant by the KS cutoff utilized. However, the graphical output showed that these strains were similar to wild type, whereas Δ*ape1* exhibited a dramatic shift in the population distribution maximum (∼2.2 standard deviations from the wild-type mean). Shape mode 3 (cell width) was significantly different in all strains compared with wild type (with the exception of Δ*ape1^C^*), and each exhibited a shift of approximately 1 S.D. in the population maximum toward a reduced width compared with the wild-type mean as reflected in the graphical output. A 2D scatterplot of measurements of each individual contour of wild type, Δ*ape1*, and Δ*ape1^C^* populations for shape modes 2 and 3 (Fig. 5*C*) likewise shows that there was a clear difference in shape for the Δ*ape1* population compared with wild type and Δ*ape1^C^*. Early-exponential phase bacteria exhibited similar population shifts as for mid-exponential phase bacteria (with the exception of shape mode 3, as cell width was not captured as a major contributor to the variance in shape for wild type at this time point). The most notable shift at early-exponential phase was observed for cell curvature (shape mode 2) in Δ*ape1* (data not shown).

##### Phenotypic Analyses Reveal the Importance of O-Acetylpeptidoglycan Esterase Activity on Various Aspects of C. jejuni Physiology

The OAP mutants were assessed for different phenotypes serving as indicators of transmission and/or colonization efficiency: motility in soft agar, biofilm formation, hydrophobicity, and sensitivity to a variety of inhibitory compounds.

**FIGURE 6.**
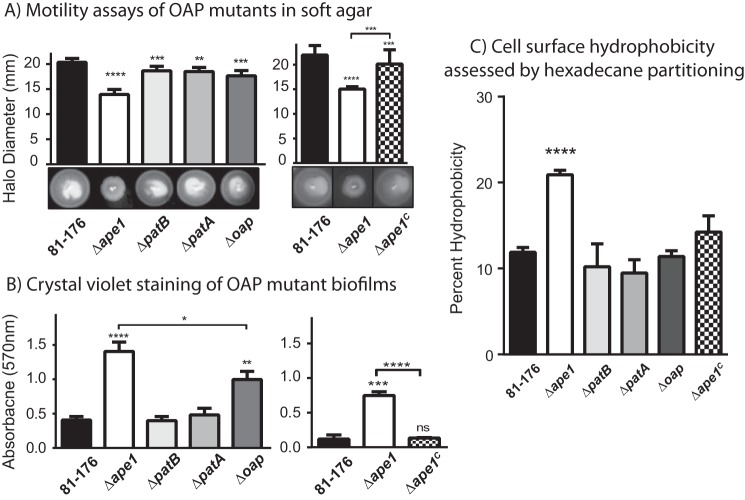
**Motility in soft agar, biofilm formation, and cell surface hydrophobicity of OAP mutants and wild-type strain 81-176.** *A,* Δ*ape1* exhibits a 30% decrease in halo diameter and abnormal halo formation (rough edges). Motility in soft agar was assessed by measuring the halo diameter after 24 h of strains point-inoculated in 0.4% semi-solid agar. Representative images of halos are shown *below* each graph. Results shown are representative of one of three independent experiments with 6 replicates. Each strain was compared with wild-type using a paired Student's *t* test, with **, ***, and **** indicating *p* < 0.01, *p* < 0.001, and *p* < 0.0001. *B*, Δ*ape1* and Δ*oap* exhibit 5.5- and 2.5-fold enhanced biofilm formation, respectively, at 24 h. Biofilm formation was assessed after 24 h by crystal violet staining of standing cultures in borosilicate tubes and spectrophotometric quantification of dissolved crystal violet at 570 nm. Results shown for the mutants (*left*) are representative of one of three independent experiments carried out in triplicate. The results for Δ*ape1^C^* (*right*) are representative of one of two experiments performed in triplicate. *ns,* not significant. *C*, Δ*ape1* exhibited a 2.0-fold increase hydrophobicity relative to wild type, as assessed by hexadecane partitioning. Results are representative of one of three independent experiments performed in triplicate. For biofilm and hydrophobicity, strains were compared using an unpaired Student's *t* test, with *, **, ***, and **** indicating *p* < 0.05, *p* < 0.01, *p* < 0.001, and *p* < 0.0001. *Error bars* represent standard deviation.

Motility is a major colonization determinant for *C. jejuni* (55). Although all strains exhibited defective halo formation compared with wild type in soft agar plates after point inoculation (Fig. 6*A*), the halo diameter of Δ*ape1* was 70% of wild type, whereas Δ*patB*, Δ*patA*, and Δ*oap* were only modestly defective at 90, 90, and 87% of wild type. Complementation of Δ*ape1* restored the halo formation of the mutant to 90% of wild type and was significantly different from that of Δ*ape1*. In addition, Δ*ape1* formed aberrant halos on soft agar with rough perimeters as opposed to the circular halos formed by wild type. This halo phenotype was absent in the other mutants tested and was rescued by complementation.

The ability to form biofilms is important in *C. jejuni* persistence and transmission. *C. jejuni* has been shown to survive up to 28 days in a biofilm state and is a general stress response (56). The ability of our OAP mutants to form biofilms was assessed in borosilicate test tubes by crystal violet staining of standing cultures (57). The Δ*ape1* mutant exhibited a hyper-biofilm phenotype, producing 5.5-fold more biofilm than wild type (Fig. 6*B*). Δ*ape1* also developed flocs of bacteria suspended in the broth (58), which were not observed for wild type nor included in the crystal violet quantification of surface-adhered biofilm (data not shown). Complementation of Δ*ape1* restored biofilm formation to wild-type levels (Fig. 6*B*). Biofilms formed by Δ*patB* and Δ*patA* were indistinguishable from wild type, but Δ*oap* produced ∼2.5-fold more biofilm than wild type. Characterization of Δ*ape1* biofilms by microscopy was unsuccessful as Δ*ape1* formed biofilms poorly on coverslips unlike wild type. This indicates altered cell surface properties in Δ*ape1*. Cell surface hydrophobicity was assessed with hexadecane partitioning (Fig. 6*C*) (59). The percent hydrophobicity of Δ*ape1* was significantly higher (2.0-fold) than wild type and was restored to wild-type levels upon complementation.

The sensitivity of the OAP mutants to detergents, salts, and antimicrobial compounds was tested by determining the minimum inhibitory concentration that reduces growth by 50% relative to a control as measured by *A*_600_ (MIC_50_) (Table 4). Only Δ*ape1* exhibited an increased susceptibility to any of the compounds tested as follows: the amphipathic bile salt sodium deoxycholate (DOC) and MgCl_2_. For Δ*ape1*, an MIC_50_ range for DOC of 0.16 to 0.31 mg/ml was observed, whereas the MIC_50_ for wild type was greater than the highest concentration of DOC tested (>10 mg/ml). Δ*ape1* exhibited a 4–8-fold reduction in MIC_50_ for MgCl_2_ compared with wild type. Complementation of Δ*ape1* restored wild type sensitivity profiles.

**TABLE 4:** **MIC_50_ of C. jejuni OAP mutants determined by broth dilution** Measurements indicated with a “-” have not been tested. Measurements in boldface were consistently ≥4-fold different from wild type over three experiments. MIC_50_, minimum inhibitory concentration to reduce growth by 50% as assessed by optical density.

Compound	MIC_50_					
	*81–176*	Δ*ape1*	Δ*patB*	Δ*patA*	Δ*oap*	Δ*ape1^C^*
**Detergents**						
Deoxycholate (mg/ml)	>10	**0.16–0.31**	5->10	1.3->10	1.3->10	1.3->10
SDS (mg/ml)	10–12.5	2.5–6.25	10–12.5	2.5–12.5	5–6.25	12.5
Triton (% v/v)	0.05	0.02–0.005	0.02–0.05	0.02–0.05	0.05	0.05

**Antimicrobials**						
Ampicillin (μg/ml)	2.4–4.9	1.2–4.9	4.9	4.9	2.4–4.9	2.4
Lysozyme (mg/ml)	>5	>5	>5	>5	>5	>5
Polymyxin B (μg/ml)	20	10	10	10	10	10–20
Protamine (μg/ml)	31.3	15.6–31.3				31.3–62.5

**Chelating agent**						
EDTA (μm)	78–156	1.2–156	156	156	156	156

**Salts**						
NaCl (mm)	62.5–250	31.3–62.5	62.5–125	125	62.5–125	62.5–125
MgCl_2_ (mm)	62.5–125	**15.6**	62.5–125	62.5–125	62.5–125	62.5–125
CaCl_2_ (mm)	125	125–250	125–250	125–500	500	125
KCl (mm)	62.5	31.3–62.5	62.5	31.3–62.5	125	31.3–62.5

##### Ape1 Is Required for C. jejuni Bacteria-Host Interactions

The contribution of OAP to *C. jejuni* host interactions was examined by determining recovery of the mutants after chick colonization and host cell infections, as well as the ability to elicit IL-8 secretion *in vitro* in human epithelial infections.

**FIGURE 7.**
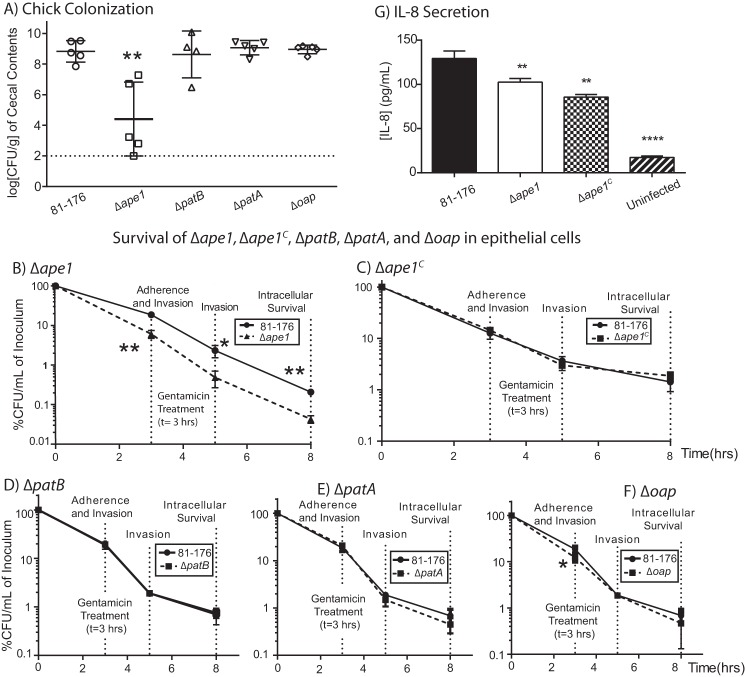
**Effect of OAP levels on *C. jejuni* host-bacteria interactions.** *A*, Δ*ape1* shows reduced chick colonization compared with wild-type strain 81-176, whereas Δ*patB*, Δ*patA*, and Δ*oap* mutants display wild-type colonization. Each point represents the recovery of *C. jejuni* strains in log CFU/g of cecal contents from individual day-old chicks 6 days post-colonization with 1 × 10^4^ CFU/ml of the indicated strain. The geometric mean is denoted by a *black bar. Error bars* represent 95% confidence intervals. Adherence, invasion, and intracellular survival of *C. jejuni* in INT407 epithelial cells were assessed by a Gm protection assay and OAP mutant strains. Δ*ape1* (*B*) shows a reduced ability to adhere to, invade, and survive in INT407 epithelial cells that were restored upon complementation (*C*). Δ*patB* (*D*), Δ*patA* (*E*), and Δ*oap* (*F*) exhibit near wild-type adherence, invasion, and intracellular survival properties. INT407 cells were infected with *C. jejuni* at a multiplicity of infection of ∼80. Adherence and invasion were quantified at 3 h post-infection. At this point, the media in the remaining wells were replaced with MEM containing gentamicin (150 μg/ml) and incubated for 2 h, after which the amount of bacterial cells that had invaded the epithelial cells was measured (5-h invasion time point). The Gm in the remaining wells was washed off, and the cells were incubated with fresh MEM containing 3% FBS and a low dose of Gm (10 μg/ml) for an additional 3 h (8-h intracellular survival time point). CFU/ml was determined for each well by lysing the cells with water and plating the dilutions onto MH-TV plates. Results for *B* and *C* are representative of three independent experiments performed in biological triplicate. The data in *D*, *E,* and *F* are representative of two independent experiments performed with three biological replicates. *G*, INT407 epithelial cells secrete less IL-8 upon infection with Δ*ape1* than wild type. Results are from one representative experiment of three independent experiments performed in triplicate. *Error bars* represent the standard deviation. *, denotes statistically significant difference using the unpaired Student's *t* test, with *, **, and **** indicating *p* < 0.05, *p* < 0.01, and *p* < 0.00001 respectively.

Chickens are an avian reservoir for *C. jejuni* and a common source of human infection. The Δ*ape1* mutant exhibited a significant 4.4-log decrease in colonization (Fig. 7*A*), whereas Δ*patB*, Δ*patA*, and Δ*oap* mutants were not defective for chick colonization. The defects in long term survival in broth for the OAP-deficient mutants were modest compared with Δ*ape1,* so the defect in chick colonization could be related to the altered long term survival properties of Δ*ape1*.

The ability of a *C. jejuni* strain to invade and survive in non-phagocytic epithelial cell lines has been shown to correlate with virulence (11, 60, 61). The ability of the *C. jejuni* OAP mutants to adhere to, invade, and survive inside the human epithelial cell line INT407 was assessed by a gentamicin (Gm) protection assay. Recovery of Δ*ape1* was significantly reduced at the adherence, invasion, and intracellular survival time points in comparison with wild type (Fig. 7*B*). Δ*ape1^C^*, Δ*patB*, Δ*patA*, and Δ*oap* displayed wild-type INT407 infection profiles (Fig. 7, *C–F*).

As Δ*ape1* was the only OAP mutant to demonstrate reduced invasion of the INT407 cells, its ability to elicit IL-8 secretion from INT407 cells was assessed by ELISA. Cells infected with Δ*ape1* reproducibly exhibited statistically significant lower levels (60–79%) of IL-8 secretion compared with cells infected with wild type (Fig. 7*G*). Δ*ape1^C^* did not complement IL-8 induction defects.

## Discussion

PG plays roles in multiple facets of bacterial physiology. PG modifications have been shown to influence pathogenic properties in several bacterial species (15, 20, 36). Here, the OAP genes in *C. jejuni* were shown to contribute to PG *O-*acetylation/de-*O*-acetylation, consistent with their predicted functions. These genes were also important for several key physiological and pathogenic properties. This was most notable for *ape1*, which was involved in PG de-*O*-acetylation and the only OAP gene significantly required for every phenotype examined.

Deletion of *patA* or *patB*, which act to *O-*acetylate MurNAc, was non-lethal as found in several other bacterial species (33, 37, 44). This suggests that *O-*acetyl groups added by PatA/B play a non-essential role for growth of *C. jejuni* in the laboratory. Unlike in *N. meningitidis* where OAP is exclusively mediated by *patA/B* (33), the *O-*acetylation levels were not reduced to 0% in *C. jejuni*, indicating the presence of alternative PG *O-*acetylation machinery or compensation by alternative mechanisms, as was observed with *E. coli* WecH that acted as an acetate transporter (62). Expression of *N. gonorrhoeae* PatB in *E. coli* increased OAP levels from <0.05 to 1%, which was detrimental to the cells (62). The effect of low levels of OAP on *E. coli* biology provides support that the residual *O-*acetylation in *C. jejuni* may be sufficient to mask mutant phenotypes in Δ*patA/B/oap* that would otherwise be observed if PG *O-*acetylation were completely absent. There are conflicting results for the essentiality of *ape1* in *N. gonorrhoeae* (43, 44). In *C. jejuni*, *ape1* was not essential. Deletion of *ape1* resulted in increased *O-*acetylation levels almost triple that of wild type, supporting the role of Ape1 in *C. jejuni* PG de-*O*-acetylation. Ape1 acetylesterase activity was also confirmed *in vitro* using the artificial substrate *p*NPAc as well as its natural substrate, *O-*acetylated PG from Δ*ape1*.

It should be noted that the OAP levels of wild-type *C. jejuni* 81-176 described here were lower than those reported in a previous study for ATCC 700819 and NCTC 11168 (42). However, the strains and growth conditions used differed between the studies. A direct comparison of how these and other potential factors might affect *C. jejuni* PG *O-*acetylation has not yet been assessed but will be the topic of future work.

As with the OAP analyses described above, the muropeptide profiles also showed differences in PG *O-*acetylation levels for the *C. jejuni* OAP mutants. Although these data are not truly quantitative for *O-*acetylation, they offer additional qualitative support for the role of these OAP genes in PG *O-*acetylation. *N. meningitidis* Δ*ape1* showed an increase in only *O-*acetylated muropeptides with a tri-peptide stem in comparison with wild type (33), although the muropeptide analysis of *C. jejuni* Δ*ape1* suggests that Ape1 in *C. jejuni* may be regulated differently, as this specificity was not observed. Previous observations with *N. gonorrhoeae* PatB *O-*acetyltransferase using *in vitro* assays showed specificity of PatB toward *O-*acetylation of tetrapeptides (30). A decrease in *O-*acetylated tetrapeptide species was observed for the *C. jejuni* Δ*patA* and Δ*patB* mutants (Table 2); however, as this could have been due to hydrolysis during the preparation procedure, further biochemical analysis will be required.

As expected, Δ*ape1* also exhibited a 42.1% decrease in relative anhMP levels (presumably due to impaired LT activity) and a greater average chain length. Although chain length was not directly measured, these data support previous findings that Ape1 regulates PG chain length as in *N. meningitidis* (33). The anhMP levels changed only marginally in Δ*patA*, Δ*patB*, and Δ*oap* supporting a putative compensation of *O-*acetylation by a yet unknown mechanism consistent with our OAP analyses and/or *O-*acetylation itself may not be an essential or the only control mechanism for LT activity in *C. jejuni*. LTs in *E. coli* and *Pseudomonas aeruginosa* have been found in complexes with peripheral membrane-bound lipoproteins and PBPs and are thought to be controlled spatially as well as coupled with synthesis to prevent autolysis (52, 63–65). For *C. jejuni*, the observations here do suggest a role for OAP in regulating LT activity, but other control mechanisms likely exist.

Differences in the muropeptide composition could be possible if *O-*acetyl groups influence substrate recognition by PG remodeling enzymes. Care must be taken in interpreting how differences in relative abundance actually affect overall PG composition. For instance, small changes in muropeptides of low abundance can result in changes ≥20% (*i.e.* total penta-Gly-5 species, which were 0.8% in wild type and 1.3% for Δ*ape1*; Table 3). Conversely, larger changes in muropeptides of high abundance can produce changes <20% yet may still be considered significant. For example, dimeric species constituted 47.7% of the muropeptides in wild type and 40.7% in Δ*ape1*; this degree of change may be meaningful, as it affects 7% of the total muropeptides, is unique compared with other mutants tested, and would be considered significant using the 10% cutoff described for *H. pylori* (27). Regardless, it is clear that the absence of *ape1* affects the PG muropeptide profile more so than the absence of *patA/B* (Table 3 and Fig. 2). These changes could be a result of increased *O-*acetylation affecting substrate recognition by PG remodeling enzymes or missing protein-protein interactions in the absence of Ape1 and will require more extensive analysis in later studies.

*N. meningitidis* Ape1 showed preference for *O-*acetylated tripeptide substrates *in vivo* (33), as mentioned above. The crystal structure for *N. meningitidis* Ape1 has recently been solved (46), confirming its classification as a member of the Ser-Gly-Asn-His (SGNH) hydrolase superfamily based on active site catalytic residues (32). These residues are also conserved in *C. jejuni* Ape1. The putative PG binding domain in the N-terminal lobe of *N. meningitidis* Ape1 and its interaction with PG have yet to be described (46). As *N. meningitidis* Ape1 was active against various *O-*acetylated muropeptides *in vitro*, specificity may be due to regulation of activity through unknown interaction partners. The putative PG binding domain at the N terminus may confer substrate specificity (33). There is only 26/42% amino acid sequence identity/similarity between the N-terminal domains of *N. meningitidis* Ape1 and *C. jejuni* Ape1, so the two enzymes may possess different regulatory regions. Another possibility is that *C. jejuni* lacks the Ape1 interaction partners present in *N. meningitidis* conferring substrate specificity.

One of *C. jejuni*'s defining characteristics is its helical shape, a trait defined by the cytoskeleton-like components that coordinate the PG biosynthetic machinery (66). The muropeptide composition was altered in *C. jejuni*/*H. pylori* periplasmic PG hydrolase mutants, *i.e.* Δ*pgp1*/Δ*csd4* and Δ*pgp2*/Δ*csd6*, and exhibited a straight rod *versus* helical morphology (18, 19, 28, 67). Deletion of *C. jejuni ape1* also resulted in altered muropeptide composition and shape, but the change in shape was not as dramatic as in the abovementioned straight-rod mutants. Thus, CellTool was employed for shape quantification. This analysis showed that Δ*ape1* was significantly different from the wild-type population in curvature in that it had both an average shape with a larger wavelength compared with wild type and a greater variance of curvature within the population. Ape1 was shown to affect cell size in *N. meningitidis* (33). In this study, there was a significant increase in total area of Δ*ape1* cells when compared with wild type at early-exponential phase but not at mid-exponential phase (data not shown). One explanation could be that Ape1 activity varies at different growth stages in *C. jejuni*.

Multiprotein flagellar complexes span the PG layer with some proteins of the complex proposed to directly interact with PG. These proteins include FlgI, which makes up the P-ring of the periplasmic rod-structure in the hook-basal body (55, 68, 69), and MotB in *H. pylori* that makes up part of the flagellar stator responsible for generating torque (70). In *Salmonella enterica*, the switch protein FliG of the C-ring, which acts as the rotary component of the flagella, responds to chemotactic signals and interacts with MotA of the stator that in turn interacts with PG-bound MotB (71). In *E. coli*, CheY is the response regulator that interacts with FliG to alter rotational direction of the flagella (72). Homologs of all these flagellar components are found in *C. jejuni* (55).

In *H. pylori*, the loss of the membrane-bound LT, MltD, affected motility without affecting the localization or number of flagella; this was hypothesized to result from the inability of MotA/B to generate torque due to impaired PG-MotB interactions (75). Similarly, the accumulation of *O-*acetylation in Δ*ape1* and the subsequent effect on LT activity could affect motility. An improperly assembled or unstable stator may impair the ability of the flagella to alter rotational direction in response to chemotactic signals. Mutants in *C. jejuni cheY* appear completely immotile on soft agar but not by microscopy (73, 74). Thus, the halo morphology does not support a complete loss in the ability to alter rotational direction but may still suggest an impaired response. The Δ*pgp1* and Δ*pgp2* straight mutants were also defective for motility in soft agar, so the changes in Δ*ape1* morphology could also account for the observed defects.

Biofilm formation in *C. jejuni* requires flagellum-mediated motility and attachment to a surface, lysis, and release of extracellular DNA to form the biofilm matrix (76). Δ*ape1* was defective for motility in soft agar but was not immotile. Despite this, Δ*ape1* exhibited a hyper- rather than hypo-biofilm formation phenotype. Envelope stress was recently shown to be a trigger for *C. jejuni* biofilm formation. A mutant exhibiting envelope stress was hyper-biofilm, and with DOC at 0.5 mg/ml, *C. jejuni* 81-176 wild type also exhibited enhanced biofilm formation (76). In this study, DOC concentrations below 0.5 mg/ml inhibit growth of Δ*ape1* (MIC_50_; Table 4). This, together with the hyper-biofilm phenotype and increased cell surface hydrophobicity, suggests that the accumulation of OAP results in altered membrane properties and may be contributing to membrane stress in Δ*ape1*. However, because Δ*oap* exhibited hyper-biofilm formation as well and did not show evidence of membrane stress (*i.e.* no change in surface hydrophobicity or DOC sensitivity), the hyper-biofilm property may be partially independent of Ape1 activity and could be due to the loss of the Ape1 protein itself.

In *E. coli*, PG-associated lipoprotein (Pal) is often found in PG-protein complexes that are proposed to maintain envelope integrity. The phenotypes of some *E. coli pal* deletion mutants share similarities to *C. jejuni* Δ*ape1,* including increased sensitivity to bile salts and signs of altered motility (77). In addition, the PG binding domain of *E. coli* MotB and *E. coli* Pal are interchangeable (78) and both interact with MurNAc; our observations in Δ*ape1* could be a result of the presence of excess of *O-*acetyl groups on the PG MurNAc residues preventing stabilizing interactions between multiprotein structures and the PG sacculus.

Given the proposed role of *O-*acetylation in lysozyme resistance, it was expected that *C. jejuni* OAP mutants would demonstrate differential resistance to lysozyme. However, no differences were observed in lysozyme sensitivity (MIC_50_). Attempts to destabilize the outer membrane by adding EDTA (at MIC_50_ and concentrations down to 4-fold less than MIC_50_) or lactoferrin (at physiological concentration of 3 mg/ml, as described previously (37)) to the lysozyme incubations also failed to result in differential lysozyme sensitivity (data not shown). Lysozyme turbidometric assays were also unsuccessful due to the low yield of PG, resulting in an initial absorbance reading too low to accurately detect a response (data not shown).

Chick colonization by Δ*ape1* was significantly impaired compared with wild type and Δ*patA*, Δ*patB,* and Δ*oap* colonized to wild-type levels (Fig. 6*A*). Motility and chemotaxis are important for colonization (79); thus, this could be a potential explanation for the Δ*ape1* chick data. Alternatively, and/or additionally, the altered morphology and PG structure (18, 19), increased DOC susceptibility, differential long term survival properties, and other as-yet unknown factors could also contribute to the Δ*ape1* colonization defect.

Of the OAP mutants, only Δ*ape1* was impaired in adherence, invasion, and intracellular survival in INT407 epithelial cells. Whether these observations represent defects at each time point, defects in adherence that in turn affect recovery at later time points, or if Δ*ape1* is very rapidly killed upon invasion (as the 3-h “adherence” time point will also reflect invaded bacteria) will require further experimentation. Infection of human INT407 epithelial cells by Δ*ape1* also led to a decrease in IL-8 secretion. This may correlate with its reduced invasion properties.

Somewhat surprisingly, the reduction in PG *O-*acetylation had no significant effects on colonization, host cell interactions, or any other phenotype examined except for marginal decreases in halo formation, suggesting that under these conditions OAP by PatA and PatB offers no fitness advantage in host survival, which is perplexing and leaves the role of PG *O-*acetylation in *C. jejuni* yet to be determined. In contrast, the increase in *O-*acetylated PG in Δ*ape1* was detrimental to *C. jejuni* in multiple aspects important for pathogenesis. Future studies will focus on finding a direct link between PG *O-*acetylation and the observed changes in physiology, identifying other potential mechanisms of PG *O-*acetylation and de-*O*-acetylation, and revealing the underlying cause(s) of the impaired host interactions for Δ*ape1*.

## Experimental Procedures

### 

#### 

##### Strains and Growth Conditions

A list of bacterial strains and plasmids used in this study can be found in Table 5. Construction of mutant and complemented strains is described in supplemental text S1 using primers listed in supplemental Table S1. *C. jejuni* strains, unless otherwise stated, were grown in Mueller-Hinton (MH; Oxoid) broth or agar (1.7% w/v) supplemented with vancomycin (10 μg/ml) and trimethoprim (5 μg/ml) and when appropriate kanamycin (Km; 50 μg/ml) and chloramphenicol (Cm; 25 μg/ml). Standard laboratory conditions for *C. jejuni* growth were 38 °C under microaerophilic conditions (12% CO_2_, 6% O_2_, in N_2_) in a Sanyo tri-gas incubator for MH agar or for standing MH broth cultures. For shaking MH broth cultures (hereafter referred to as broth cultures), *C. jejuni* were cultured in airtight jars using the Oxoid CampyGen Atmosphere generation system with shaking at 200 rpm. Experiments were performed using cultures initiated at *A*_600_ 0.002 and grown in shaking broth for 16–18 h to reach exponential phased. For plasmid construction and protein purification, *E. coli* (DH5-α or BL21) strains were grown at 37 °C in Luria-Bertani (LB; Sigma) broth or LB agar (7.5% w/v) supplemented with ampicillin (100 μg/ml), Km (25 μg/ml), or Cm (15 μg/ml) as required.

**TABLE 5:** **Bacterial strains or plasmids used in this study**

Strain or plasmid	Genotype or description	Source
***C. jejuni* strains**		
81-176	Wild-type isolated from diarrheic patient	87
Δ*ape1*	81-176 *ape1*::*aphA3*;Km^R^	This study
Δ*patB*	81-176 *patB*::*aphA3*;Km^R^	This study
Δ*patA*	81-176 *patA*::*aphA3*;Km^R^	This study
Δ*oap*	81-176 *oap*::*aphA3*;Km^R^	This study
Δ*ape1^C^*	81-176 Δ*ape1 rrn*::*ape1* (from pRRC-*0638*)	This study
Δ*patB^C^*	81-176 Δ*patB rrn*::*patB* (from pRRC-*0639*)	This study
Δ*patA^C^*	81-176 Δ*patA rrn*::*patA* (from pRRC-*0640*)	This study

***E. coli* strains**		
DH5-α	F^−^, ϕ80d *deoR lacZ*Δ*M15 endA1 recA1 hsdR17*(r_K_-m_K_+) *supE44 thi-1 gyrA96 relA1* Δ*(lacZYA-argF) U169*	Invitrogen
BL21(λDE3)	F^−^ omp*T* hsd*S_B_*(r_B_^−^, m_B_^−^) gal dcm [λ DE3]	Novagen

**Plasmids**		
pGEM-T	High copy, linearized, T-tailed, Blue/White, Ap^R^	Promega
pUC18-K2	Source of non-polar *aphA3* cassette; Ap^R^Km^R^	47
pGEM-T-*0638*	pGEM-T ligated to 0638 amplified with 0637-2 and 0639-5 (2113 bp); Ap^R^	This study
pGEM-*0638*::*aphA-3*	pGEM-T-*0638* inverse PCR amplified with 0638-3 and 0638-2 (4098 bp) and ligated to *aphA-3* (KpnI, HincII); Ap^R^, Km^R^	This study
pGEM-T-*0639*	pGEM-T ligated to 0639 amplified with 0639-1 and 0639-2 (2146 bp); Ap^R^	This study
pGEM-*0639*::*aphA-3*	pGEM-T-*0639* inverse PCR amplified with 0639-3 and 0639-4 (4267 bp) and ligated to *aphA-3* (KpnI, HincII); Ap^R^, Km^R^	This study
pGEM-T-*0640*	pGEM-T ligated to 0640 amplified with 0639-6 and 0641-4 (2396 bp); Ap^R^	This study
pGEM-*0640*::*aphA-3*	pGEM-T-*0640* inverse PCR amplified with 0640-1 and 0640-2 (4168 bp) and ligated to *aphA-3* (KpnI, HincII); Ap^R^, Km^R^	This study
pGEM-*0638–40*::*aphA-3*	pGEM-T ligated to 0637 fragment amplified with 0637-1 and 0638-1 (1483 bp), 0641 fragment amplified with 0641-1 and 0641-2 (822 bp), and *aphA-3* (KpnI, HincII); Ap^R^, Km^R^	This study
pRRC	*C. jejuni* rRNA spacer integration vector; Cm^R^	48
pRRC-*0638*	pRRC ligated to *0638* amplified with 0638-C1(NheI) and 0638-C2(MfeI) (1347 bp); Cm^R^	This study
pRRC-*0639*	pRRC ligated to *0639* amplified with 0639-C1(NheI) and 0639-C2(MfeI) (1276 bp); Cm^R^	This study
pRRC-*0640*	pRRC ligated to *0640* amplified with 0640-C1(NheI) and 0640-C2(MfeI) (1616 bp); Cm^R^	This study
pET28a(+)	Commercial vector for expression of recombinant His_6_-tagged protein	Novagen
p0638-His_6_	pET28a(+) ligated to *ape1* amplified with 0638-eCF (NcoI) and 0638-eCR (EcoRI) (1121 bp) for expression of C-terminal His_6_-tagged 0638; Km^R^	This study
pHis_6_-0638	pET28a(+) ligated to *ape1* amplified with 0638-eNF (NheI) and 0638-eNR (EcoRI) (1116 bp); for expression of N-terminal His_6_-tagged 0638 Km^R^	This study

##### PG Isolation and Assessment of O-Acetylation Levels

PG isolation for *O-*acetylation analysis was performed as described previously with minor modifications (32, 51). Each strain was grown on ∼60 MH-T agar plates (supplemented with Km or Cm as necessary) for ∼18–20 h. The cells were harvested from the plate with 1 ml of ice-cold MH broth per plate and added to a conical tube. Strains were assessed by DICM to examine for contamination and the presence of coccoid cells, ensuring that the cultures had not entered stationary phase. *C. jejuni* cells transition from a helical to coccoid form in stationary phase. The cells were collected by centrifugation, resuspended in 50 ml of 25 mm sodium phosphate buffer, pH 6.5, and boiled in an equal volume of 8% w/v SDS in 25 mm sodium phosphate buffered at pH ∼6.5 for 3 h under reflux with stirring (final concentration 4% SDS w/v). SDS-insoluble PG was washed with sterile double distilled H_2_O (methylene blue/chloroform tests were performed to detect SDS) (80), frozen, and lyophilized. Lyophilized PG was resuspended in a minimal volume of buffer containing 10 mm Tris-HCl, pH 6.5, and 10 mm NaCl and sonicated (Misonix XL 2020, Mandel Scientific) on ice with a microtip for 2 min. The suspension was treated with 100 μg/ml α-amylase (Fluka Biochemika), 10 μg/ml DNase I (Invitrogen), 50 μg/ml RNase A (ThermoScientific), and 20 mm MgSO_4_ overnight at 37 °C. Protease (from *Streptomyces griseus*, Sigma), pre-incubated at 60 °C for 2 h, was added to 200 μg/ml and incubated overnight at 37 °C. Samples were then re-extracted in SDS, purified as above, lyophilized, and stored at −20 °C. *O-*Acetylation levels of lyophilized PG were evaluated as a ratio of total saponified *O-*linked acetate relative to total MurNAc content using mild base-catalyzed release of *O-*linked acetate (0.1 m NaOH, 40 °C for 4 h) and acid-catalyzed hydrolysis (6 n HCl, 100 °C for 1.5 h) for the complete liberation of PG monosaccharides, acetate, and muramic acid content. Components were quantified by HPLC as described previously (49, 50).

##### PG Isolation and Muropeptide Analysis

Each strain was grown on ∼20–25 MH-T plates (supplemented with Km as required) for ∼18–20 h to standardize growth phase and harvested with ice-cold MH-TV broth. Strains were assessed by DICM for contamination and coccoid cells to ensure that cultures had not grown into stationary phase. Cells were lysed using the boiling SDS technique as described previously (19). PG was further purified from the cell lysate and digested with the muramidase cellosyl (kindly provided by Hoechst, Frankfurt, Germany). The muropeptides were reduced with sodium borohydride, and subsequently separated by HPLC, all as described previously (81). Muropeptide structures were assigned based on (i) comparison with retention times of known muropeptides from *C. jejuni* and (ii) by mass spectrometry (Fig. 2) (18, 19, 82).

##### Expression, Purification, and Biochemical Assays of C. jejuni Ape1-His_6_

*Cjj81176_0638* (encoding *ape1*) was PCR-amplified without the predicted 21-amino acid signal peptide (as identified by SignalP 4.1 Server) (83) and cloned into the pET28a(+) (Novagen) expression vector in-frame with the encoded His_6_ tag at either the N or C termini of the gene forming pHis_6_-0638 and p0638-His_6_, respectively (Fig. 3, *A* and *B*). Expression constructs were transformed into *E. coli* BL21(λDE3), selected for Km^R^, and confirmed by PCR and sequencing. Expression and isolation of recombinant Ape1 are described in the supplemental text S2.

Acetylesterase activity was determined using *p*NPAc as a colorimetric substrate, as described previously (45). Assays were performed with 2.5 μg/ml purified Ape1-His_6_ in 50 mm sodium phosphate buffer, pH 6.5, and 2 mm *p*NPAc. Reactions were monitored over 5 min, and specific activity was calculated with an experimentally determined molar absorptivity of 3.42 mm^−1^ cm^−1^ for *p*-nitrophenol (room temperature at pH 6.5).

PG-*O*-acetylesterase activity was tested using purified PG retaining *O-*acetyl groups (as described under “PG Isolation and Assessment of *O*-Acetylation Levels”). Lyophilized PG was resuspended in sodium phosphate buffer, pH 6.5, to a concentration of 5 mg/ml and sonicated on ice with a microtip for 2 min (10 s on/10 s off). 500 μl of PG suspension was aliquoted into Eppendorf tubes to which, Ape1-His_6_ was added to a concentration of 10 μg/ml (buffer only for negative control). Samples were incubated at 37 °C in a water bath for 24 h, after which samples were centrifuged (10 000 × *g*, 10 min, 4 °C) to pellet PG. Acetate content in supernatants were assessed using a commercial acetic acid assay kit (Megazyme International) as directed by the manufacturer.

##### Microscopy and CellTool Shape Analysis

Overnight MH-TV log-phase broth cultures were standardized to *A*_600_ 0.05 and incubated for 4 or 7 h at 38 °C to generate early-exponential phase and mid-exponential phase cultures, respectively. The samples were processed for DICM. Live cells were imaged on agarose slabs on a Nikon Eclipse TE2000-U microscope equipped with a Hamamatsu C4742-95 digital camera.

For CellTool analysis (53), DICM images from multiple fields (yielding ≥400 cells per strain) were taken for each sample and processed by thresholding to generate binary images. Artifacts and cells that were clumped or ill-represented based on lighting were manually removed. The contours of the wild-type population were aligned to generate an average shape, and PCA was performed to generate a “shape model” based off principal components called “shape modes” that, together, describe at least 95% of the variation in the wild-type population. Contours of other strains were then aligned to the wild-type PCA shape model as a reference. Kolmogorov-Smirnov tests were used on each shape mode to determine whether there was a statistically significant difference in population distribution between the strains based on this wild-type shape model (53).

##### Phenotypic Characterization of OAP Mutants: Motility, Biofilm, Minimum Inhibitory/Bactericidal Concentrations and Cell Surface Hydrophobicity

Motility and biofilm formation assays were performed on log-phase bacterial broth cultures as described previously (19).

MIC_50_ was determined in a 96-well plate as standing culture as described previously (84). Inocula of log-phase overnight cultures (100 μl) standardized to *A*_600_ 0.0002 (10^6^ CFU/ml) in MH-TV and 11 μl of 10× concentrated test compound (in 2× serial dilutions) were added to each well. *A*_600_ was measured for each well using the Varioskan Flash Multimode Plate Reader (Thermo Scientific), and MIC_50_ was recorded as the lowest concentration of compound that reduced growth by 50% (by turbidity) relative to a positive control after 24 h.

Cell surface hydrophobicity was assessed using exponential phase bacterial broth cultures as described previously (85) with the following adjustments. Cultures were harvested at 8 000 × *g* for 10 min and washed three times with PBS. Cells were resuspended to an *A*_600_ ∼0.5 in PBS, and the absorbance was recorded. Hexadecane was added to the standardized cultures in a ratio of 1:4 hexadecane/culture by volume, vortexed for 5 min, and incubated at 38 °C for 30 min. The aqueous layer was isolated, aerated by bubbling N_2_ gas through the aqueous layer for 30 s, and left open to the air for 10 min to ensure removal of all traces of hexadecane, and the *A*_600_ was measured. Cell surface hydrophobicity was expressed as follows, where *A*_600_*i* and *A*_600_*f* refer to the optical densities before and after extraction, respectively.

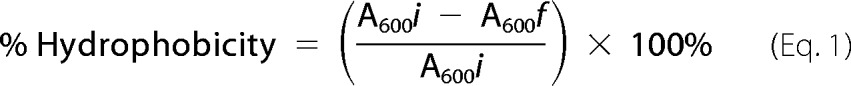


##### Chick Colonization

Chick colonization was assessed under protocol 10462 approved by the University of Michigan Committee on Care and Use of Animals, as described previously (19, 79).

##### Gentamicin Protection Assay for Host-Cell Infection

Gm protection assays were performed essentially as described previously (86). INT407 human epithelial cells were seeded into 24-well tissue culture plates at ∼1.25 × 10^5^ cells in minimum essential medium (MEM) supplemented with 10% (v/v) FBS and 1× penicillin/streptomycin (Gibco, Life Technologies, Inc.) 24 h prior to infection. Infections were initiated by adding log-phase bacterial cultures standardized to *A*_600_ 0.002 in MEM (1 × 10^7^ CFU/ml) and added to INT407 cells previously washed twice with 1 ml of MEM to give a multiplicity of infection of ∼80. Adherence/invasion after 3 h of infection, invasion following a 2-h Gm treatment (150 μg/ml) to kill extracellular bacteria, and intracellular survival following removal of the high Gm concentration and incubation of cells in fresh medium with 10 μg/ml Gm and 3% FBS for an additional 3 h were assessed as described (86).

##### Interleukin-8 Quantification

The concentration of IL-8 secreted by INT407 cells either left uninfected or infected with *C. jejuni* wild-type strain 81-176, Δ*ape1*, or Δ*ape1^C^* for 24 h was assayed using the human IL-8 ELISA kit (Thermo Fisher Scientific) as described previously (18).

## Author Contributions

Initial conceptualization of the project details was by E. F. and E. C. G. R. H. conducted most of the experiments in the study and prepared the manuscript with E. C. G. and E. F. D. S conducted the *O-*acetylation assays on purified PG from OAP mutants. J. B. conducted experiments on the muropeptide profiles of the OAP mutants. M. E. T. and J. G. J. conducted the chick colonization assays with OAP mutants. Oversight of the project was provided by E. C. G., A. J. C., W. V., and V. J. D. All authors reviewed and approved the final version of the manuscript.

## Supplementary Material

Supplemental Data

## Footnotes

6 The abbreviations used are:

PG: peptidoglycan
OAP: *O-*acetylation of peptidoglycan
MurNAc: *N*-acetylmuramic acid
LT: lytic transglycosylase
anhMP: anhydromuropeptide
*p*NPAc: *p*-nitrophenyl acetate
DICM: differential interference contrast microscopy
PCA: principal component analysis
MEM: minimum essential medium
DOC: deoxycholate
Gm: gentamicin
KS: Kolmogorov-Smirnov
MH: Mueller-Hinton
Cm: chloramphenicol
Km: kanamycin.
