# Supplementary material for: Accumulation of Peptidoglycan O-Acetylation Leads to Altered Cell Wall Biochemistry and Negatively Impacts Pathogenesis Factors of Campylobacter jejuni
Source: J Biol Chem. 2016 Jul 29;291(43):22686–702. doi: 10.1074/jbc.M116.746404 (PMC5077204; doi:10.1074/jbc.M116.746404)
Supplement: Supplemental Data [file supp_291_43_22686__index.html]

Accumulation of Peptidoglycan O-Acetylation Leads to Altered Cell Wall Biochemistry and Negatively Impacts Pathogenesis Factors of Campylobacter jejuni — Accumulation of Peptidoglycan O-Acetylation Leads to Altered Cell Wall Biochemistry and Negatively Impacts Pathogenesis Factors of Campylobacter jejuni — Role of C. jejuni Peptidoglycan O-Acetylation — Supplemental Data 

# Accumulation of Peptidoglycan *O*-Acetylation Leads to Altered Cell Wall Biochemistry and Negatively Impacts Pathogenesis Factors of *Campylobacter jejuni*

## Supplemental Data

- Supplemental text and data (.pdf, 331 KB) - Detailed description of cloning methods used to construct mutants, complemented strains, and expression constructs, as well as a list of primers used.
