# Supplementary material for: Accumulation of Peptidoglycan O-Acetylation Leads to Altered Cell Wall Biochemistry and Negatively Impacts Pathogenesis Factors of Campylobacter jejuni
Source: J Biol Chem. 2016 Jul 29;291(43):22686–702. doi: 10.1074/jbc.M116.746404 (PMC5077204; doi:10.1074/jbc.M116.746404)
Supplement: Supplemental Data [file 10.1074_M116.746404_jbc.M116.746404-1.pdf]

Accumulation of Peptidoglycan O-Acetylation leads to Altered Cell Wall Biochemistry and Negatively Impacts Pathogenesis Factors of *Campylobacter jejuni**

Supplemental Text and Data

Reuben Ha[1], Emilisa Frirdich[1], David Sychantha[2], Jacob Biboy[3], Michael E. Taveirne[4,5], Jeremiah G. Johnson[4,6], Victor J. DiRita[4,6], Waldemar Vollmer[3], Anthony J. Clarke[2], Erin C. Gaynor[1]
From the [1]Department of Microbiology and Immunology, University of British Columbia, Vancouver, British Columbia V6T 1Z3, Canada; the [2]Department of Molecular and Cellular Biology, University of Guelph, Guelph, Ontario N1G 2W1, Canada; the [3]Centre for Bacterial Cell Biology, Institute for Cell and Molecular Biosciences, Newcastle University, Newcastle upon Tyne NE2 4AX, United Kingdom; and the [4]Department of Microbiology and Immunology, University of Michigan Medical School, Ann Arbor, Michigan 48109; [5]Current address: Department of Biological Sciences, North Carolina State University, Raleigh NC 27695; [6]Current address: Department of Microbiology & Molecular Genetics, Michigan State University, East Lansing, MI 48824

Text S1. Deletion and complementation of OAP mutants

Genes were PCR amplified using iProof™ (Bio-Rad) with primers designed to include 500-600 bp of flanking DNA on either side of each gene (for PCR primers used for each gene, refer to Table S1). PCR products were adenylated at the 3'-ends using Taq DNA Polymerase (Invitrogen) and ligated to commercial cloning vector pGEM-T (Promega), resulting in pGEM-T-*0638*, pGEM-T-*0639*, and pGEM-T-*0640*. Vectors were transformed into *E.coli* DH5-α and selected for Ap^R resistance and blue/white screening. All constructs were verified by sequencing and restriction digestion. The resulting constructs were inverse PCR amplified using primers designed to amplify the flanking regions and pGEM-T vector without the target gene save for the sequences used for primer design at the 5' and 3' ends of each gene (for PCR primers used for each gene, refer to Table S1).Approximately 86% of *cjj81176_0638*, 87% of *cjj81176_0639*, and 89% of *cjj81176_0640* were deleted by inverse PCR. Inverse products were restriction digested where appropriate with sites encoded within the primers and ligated to the non-polar Km^R cassette (*aphA-3*) digested out of pUC18K-2 (1) with *KpnI* and *HincII* to form the deletion-constructs pGEM-*0638::aphA-3*, pGEM-*0639::aphA-3*, and pGEM-*0640::aphA-3*. To generate pGEM-*0638-40::aphA*-3 deletion construct, the primers 0637-1/0638-1(*KpnI*) and 0641-1(*NcoI*)/0641-2(*KpnI*) were used to amplify the regions downstream and upstream of the OAP operon, respectively. The downstream PCR product was adenylated, ligated to pGEM-T, digested with *KpnI* and *NcoI* and ligated to the upstream PCR product (digested with *KpnI* and *NcoI*) generating pGEM-T-0637+0641. The *aphA-3* from pUC18K-2 (*KpnI* and *HincII* digested) was ligated to pGEM-T-0637+0641 digested with *KpnI* and *SmaI* resulting in pGEM-T-0638-40Km^R and led to a deletion construct in which 98% of the putative *oap* operon was replaced by *aphA-3*. *C. jejuni* 81-176 was naturally transformed with each deletion construct. Mutant strains were selected by Km^R and verified by PCR, restriction digestion, and sequencing.

Genes were complemented using the pRRC system for gene delivery and expression (2). The genes *cjj81176_0638*, *cjj81176_0639*, and *cjj81176_0640* were each individually PCR amplified using the complementation primers (denoted with a C in the primer name after the gene designation in Table S1). PCR products and pRRC vectors were restriction digested with *MfeI/NheI* and *XbaI/NheI*, respectively and ligated together. Resulting complementation constructs were transformed into *E.coli* DH5α via heat shock, selected for resistance to Cm^R on LB-Cm^R agar plates and confirmed by PCR and sequencing. Complementation constructs were naturally transformed into their respective mutants with the exception of pRRC-*0638* which was first methylated before transformation into Δ*ape1*. Successful transformants were confirmed by PCR (Cat-2 and corresponding reverse complement primers designated

with C2, Table S1) (3) and the rRNA locus of each insertion was confirmed using ak233, ak234 and ak235 primers specific to each of the three ribosomal spacer regions combined with the C2 primers (Table S1) (2).

Text S2. Cloning of Ape1 for Expression and Purification

The *ape1* gene was PCR amplified using primer pairs 0638-His-NF/0638-His-NR and 0638-His-CF/0638-His-CR for insertion into pET28a(+) in frame with the encoded N-terminal and C-terminal 6xHis tags, respectively to yield pHis$_6$-0638 and p0638-His$_6$ respectively. Primer 0638-His-NF was designed with an *NheI* restriction site for insertion downstream of pET28a(+) encoded 6xHis tag and thrombin cleavage site and will amplify *cjj81176_0638* starting after the signal sequence predicted by SignalP 4.1. Primer 0638-His-NR is designed with an *EcoRI* restriction site for cloning into the MCS and contains the originally coded TAA stop codon. Primer 0638-His-CF was designed with an *NcoI* restriction site, that contains an AUG start codon and will amplify *cjj81176_0638* starting after the signal sequence predicted by SignalP 4.1. Primer 0638-His-CR is designed with an *EcoRI* restriction site for cloning into the MCS and does not encode a stop codon allowing for translation through to the 6xHis tag followed by a TGA stop codon encoded in the pET28a(+) expression vector.

Expression vectors were transformed into *E. coli* BL21 for expression. BL21-pET28a(+), BL21-pHis$_6$-0638 , and BL21- p0638-His$_6$were grown overnight under standard conditions with Km, as required. Strains from overnight cultures were subcultured to an OD600 of 0.2 and grown to OD_{600} of ~0.5-0.6 before induction with 1 mM IPTG at room temperature for 3 hrs. Bacterial lysates were prepared by sonication for 2 mins (10s On/ 10 s OFF) in Lysis Buffer 1 (50 mM sodium phosphate buffer pH~8.0, 300 mM NaCl, 10 mM imidazole) and recombinant Ape1 was purified by Ni-NTA agarose (Qiagen) affinity chromatography. The sample was washed twice with 4 mL of Wash Buffer 1 (20 mM imidazole, 50 mM sodium phosphate buffer pH 8.0, 300 mM NaCl), once with 4 mL of Wash Buffer 2 (35 mM imidazole, 50 mM sodium phosphate buffer pH 8.0, 300 mM NaCl), and eluted with elution buffer (200 mM imidazole, 50 mM sodium phosphate buffer pH 8.0, 300 mM NaCl). Purified protein was dialyzed against 250 x sample volume of dialysis buffer (50 mM sodium phosphate buffer pH 8.0, 30 mM NaCl) using Slide-A-Lyzer (ThermoScientific) at 4 ^oC. The dialysis buffer was changed twice over 36 hrs. The sample was recovered and any precipitate present was removed via centrifugation. Quantification of total soluble protein was performed with Bio-Rad Dc Protein Assay Kit or by measuring the absorbance at 280 nm on a Nanodrop (ND-1000, ThermoScientific). Samples were concentrated using 3K MWCO (Millipore) as directed and concentrated samples were flash frozen in liquid N_2 as 10μL aliquots for storage at -80°C until required.

Table S1. List of Primers used in this study with underlined restriction sites in lowercase

Primer[1] (Source)	Sequence (5' to 3')	Restriction Site
**Δ*ape1***		
0637-2	GGTGGAGCATTAAGCGGAA	
0639-5	GCCACCAATAAGGCTTTTATTAAAAACTC	
0638-2*	GAAggtaccTGAGTATGGAATCTATTGTGTCATTTGTATTTA	*KpnI*
0638-3*	CTATGAGCTGATGGCTAAAAAGTTACT	
**Δ*patB***		
0639-1	GGCTAGATGGCATATCAGTCTTTC	
0639-2	GACCATTTGTTAATTTGTGTAGTTCTTAACTC	
0639-3*	GAAggtaccCTACAACAACCAAGCCAAGCA	*KpnI*
0639-4*	GTGGCGCAAGAGAAATGTCT	
**Δ*patA***		
0639-6	GACCAGTCAAAATAGGATTTATAAGAAAGG	
0641-4	GCAGCAAACTTATATCTTAGTATGAGTTCTT	
0640-2 *	GAAggtaccGCTATCATTAAAATACTAAATTCTAGAGAAAAATAAGTC	*KpnI*
0640-1*	GAATCTCCATTTTTACTAAAACCTTTTATCATAGC	
**Δ*oap***		
0637-1	TGCAAGAAATGCAGCAAATTTAAATCC	
0638-1	GAAggtaccCTATGAGCTGATGGCTAAAAAGTTACT	*KpnI*
0641-1	GGATACACAGTCGATGAACCTG	*NcoI*
0641-2	GAAggtaccAcccgggCCTTCAATCCAAAGCGAGATTTG	*KpnI, SmaI*
**Resistance Markers**		
cat-2 (3)	GTTTTTTGGATGAATTACAAGA	
aphA3-2 (3)	CTATTTTTTGACTTACTGGGGA	
**Complement**		
0638-C1	GCGGCGgctagcCGCAAGAGAAATGTCTAAACTTTTATTAGAAC	*NheI*
0638-C2	GCGGCGcaattgCCCAGCAAGAATTTTTAAGAGCTAGATT	*MfeI*
0639-C1	GCGGCGgctagcCTTTTGGGGTTCTTTTTATGATCTATCCTTTA	*NheI*
0639-C2	GCGGCGcaattgTGAGTATGGAATCTATTGTGTCATTTGTATTTA	*MfeI*
0640-C1	GCGGCGgctagcGGTATGTAAGCGAACAACACGAA	*NheI*
0640-C2	GCGGCGcaattgACCAAGCCAAGCACTATAATCAAAATAA	*MfeI*
**Ribosomal 16S Markers**		
ak233 (2)	GCAAGAGTTTTGCTTATGTTAGCAC	
ak234 (2)	GAAATGGGCAGAGTGTATTCTCCG	
ak235 (2)	GTGCGGATAATGTTGTTTCTG	
**Protein Expression**		
0638-His-NF	GCGGCGgctagcCAAAATTTAAATACAAATGACACAATAGATTCCATACT	*NheI*
0638-His-NR	GCGGCGgaattcTTAATAATCAATGATATTTTTTAAATCCTCGAGTAACTT	*EcoRI*
0638-His-CF	GCGGCGccatggGGCAAAATTTAAATACAAATGACACAATAGA	*NcoI*

	TTCCATACT	
0638-His-CR	GCGGCGgaattcCCATAATCAATGATATTTTTTAAATCCTCGAGT AACTT	*EcoRI*

[1] An asterisk (*) after the primer name indicates primers used for inverse PCR amplification for generating deletion constructs. The letter **C** in the primer name indicates primers used to generate **c**omplementation constructs in pRRC vectors. His in the primer name indicates primers used to amplify *Cjj81176_0638* for generating His-tagged protein **e**xpression constructs.

References:

1. Menard, R., Sansonetti, P. J., and Parsot, C. (1993) Nonpolar mutagenesis of the ipa genes defines IpaB, IpaC, and IpaD as effectors of Shigella flexneri entry into epithelial cells. *J. Bacteriol.* **175**, 5899-5906
2. Karlyshev, A. V., and Wren, B. W. (2005) Development and application of an Insertional system for gene delivery and expression in Campylobacter jejuni. *Appl. Environ. Microbiol.* **71**, 4004-4013
3. Frirdich, E., Biboy, J., Adams, C., Lee, J., Ellermeier, J., Gielda, L. D., DiRita, V. J., Girardin, S. E., Vollmer, W., and Gaynor, E. C. (2012) Peptidoglycan-Modifying Enzyme Pgp1 Is Required for Helical Cell Shape and Pathogenicity Traits in Campylobacter jejuni. *PLoS Path.* **8**
